# Microarray scanner calibration curves: characteristics and implications

**DOI:** 10.1186/1471-2105-6-S2-S11

**Published:** 2005-07-15

**Authors:** Leming Shi, Weida Tong, Zhenqiang Su, Tao Han, Jing Han, Raj K Puri, Hong Fang, Felix W Frueh, Federico M Goodsaid, Lei Guo, William S Branham, James J Chen, Z Alex Xu, Stephen C Harris, Huixiao Hong, Qian Xie, Roger G Perkins, James C Fuscoe

**Affiliations:** 1 National Center for Toxicological Research, U.S. Food and Drug Administration, 3900 NCTR Road, Jefferson, Arkansas 72079, USA; 2 Center for Biologics Evaluation and Research, U.S. Food and Drug Administration, NIH Campus Building 29B, 29 Lincoln Drive, Bethesda, Maryland 20892, USA; 3 Z-Tech Corporation, 3900 NCTR Road, Jefferson, Arkansas 72079, USA; 4 Center for Drug Evaluation and Research, U.S. Food and Drug Administration, 1451 Rockville Pike, Rockville, Maryland 20852, USA

## Abstract

**Background:**

Microarray-based measurement of mRNA abundance assumes a linear relationship between the fluorescence intensity and the dye concentration. In reality, however, the calibration curve can be nonlinear.

**Results:**

By scanning a microarray scanner calibration slide containing known concentrations of fluorescent dyes under 18 PMT gains, we were able to evaluate the differences in calibration characteristics of Cy5 and Cy3. First, the calibration curve for the same dye under the same PMT gain is nonlinear at both the high and low intensity ends. Second, the degree of nonlinearity of the calibration curve depends on the PMT gain. Third, the two PMTs (for Cy5 and Cy3) behave differently even under the same gain. Fourth, the background intensity for the Cy3 channel is higher than that for the Cy5 channel. The impact of such characteristics on the accuracy and reproducibility of measured mRNA abundance and the calculated ratios was demonstrated. Combined with simulation results, we provided explanations to the existence of ratio underestimation, intensity-dependence of ratio bias, and anti-correlation of ratios in dye-swap replicates. We further demonstrated that although Lowess normalization effectively eliminates the intensity-dependence of ratio bias, the systematic deviation from true ratios largely remained. A method of calculating ratios based on concentrations estimated from the calibration curves was proposed for correcting ratio bias.

**Conclusion:**

It is preferable to scan microarray slides at fixed, optimal gain settings under which the linearity between concentration and intensity is maximized. Although normalization methods improve reproducibility of microarray measurements, they appear less effective in improving accuracy.

## Background

The reliability of microarray data is dependent on many factors including the performance of the signal readout system [1-3]. Fluorescence is currently the predominant method for microarray signal detection not only for two-color systems but also for most one-color systems [4-6]. A critical component of a fluorescence scanner is the photomultiplier tube (PMT), in which fluorescent photons produce electrons that are amplified by the PMT voltage, also referred to as the PMT gain. For many microarray scanners, the PMT gain is an easily adjustable parameter, and the calibration curve (*i.e*., the curve showing the relationship between dye concentration and fluorescence intensity) depends on the gain setting [5-7].

DNA microarray measurements normally assume a linear relationship between detected fluorescent signal and the concentration of the fluorescent dye that is incorporated into the cDNA or cRNA molecules synthesized from the test sample. Each PMT has its own linear dynamic range within which signal intensity increases linearly with the increase of fluorescent dye concentration [5,6]. However, due to the wide concentration range for genes expressed in a biological sample, the detected fluorescence intensity does not necessarily remain in the linear range for all genes tiled on a microarray. In addition, the background fluorescence intensity of the Cy3 channel is generally higher than that of the Cy5 channel [8-10]. Nonlinearity between fluorescence intensity and dye concentration can occur due to chemical saturation, dye quenching, signal bleaching, optical saturation, and instrument limitations. The impact of such nonlinearity on microarray data accuracy and reproducibility has been suggested and normalization methods have been proposed for correcting systematic and nonlinear bias. Lowess (locally weighted scatter plot smooth) is a locally weighted linear regression method that has been proposed and widely accepted as a normalization method for correcting intensity-dependent ratio bias [11-13].

Most studies dealing with nonlinearity in microarray data have been focusing on the intensity space, *i.e*., correcting the nonlinear relationship between intensity data from different PMT gains. For example, Dudley *et al*. applied a linear regression method on data acquired from the same slide under several PMT gains to extend the linear range of a scanner [14]. Similar strategies of scanning the same slide at multiple PMT gains to extend the dynamic range of intensity have been reported by others [15-18].

In this study, we evaluate the characteristics and implications of the calibration curves for the two commonly used dyes (Cy5 and Cy3) under different PMT gains and offer explanations for several experimental observations commonly encountered in two-color microarray platforms. The effectiveness of Lowess and mean normalization methods on the accuracy and reproducibility of ratios estimated by microarray technology is assessed. A method of calculating ratios based on concentrations estimated from the calibration curves is proposed for correcting ratio bias. To our knowledge, the current work represents the most comprehensive study investigating the calibration characteristics and implications of the Cy5 and Cy3 under various PMT gain settings.

## Methods

### Microarray scanner calibration slide

**Figure 1:**
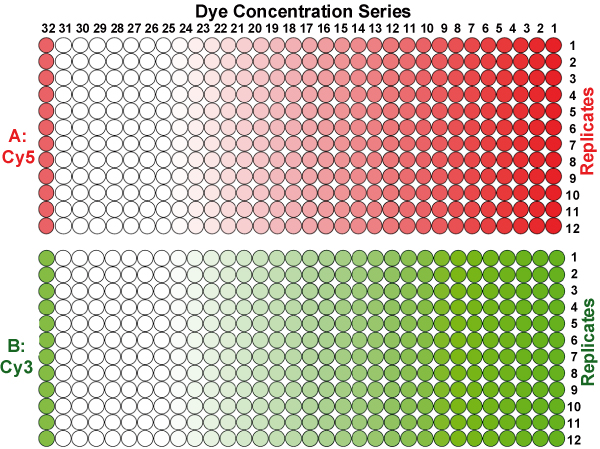
**The layout of a microarray scanner calibration slide. **A: Cy5 block; B: Cy3 block. There are 384 (32 by 12) spots for each of the Cy5 or Cy3 block. Series 1 to 28 correspond to two-fold dilutions, each of which has 12 repeats. Series 29 to 31 are blanks and series 32 is the positioning marker.

The microarray scanner calibration slide from Full Moon BioSystems Inc. (Sunnyvale, California, USA) has been developed for performing quantitative evaluations of microarray scanners in terms of dynamic range, limit of detection, uniformity of microarray scanners, channel-to-channel cross-talk, and laser stability. Details can be found at  and [6]. The array layout of the calibration slide is shown in Figure 1. Briefly, on a specially treated glass slide (1" by 3") two separate blocks of arrays in dilution series of Cy5 (Block A of Figure 1) and Cy3 (Block B of Figure 1) fluorescent dyes are spotted. Each block consists of 28 sets of two-fold dilutions of Cy3 or Cy5 (1–28), coupled with 3 sets of blanks (29–31) and one set of position markers (32). Each column contains 12 repeats of each sample (concentration). This scanner calibration slide allows us to separate the characteristics of the fluorescent dyes and the photomultiplier tubes from other factors such as labelling and hybridization. Dye concentration is expressed as fluorophores/μm^2^. The highest and lowest concentrations are 1.47 × 10^5 ^fluorophores/μm^2 ^(for series #1) and 1.10 × 10^-3 ^fluorophores/μm^2 ^(for series #28), respectively. In the calculation of log intensity correlation and log ratio correlation, only 14 dilution series (#6 to #19) are used, corresponding to a concentration difference of 4096-fold.

### Spotting oligonucleotide microarrays

Mouse 20 K oligonucleotides from MWG Biotech (High Point, North Carolina, USA) were spotted on glass slides as described elsewhere [19].

### Microarray labeling and hybridization reactions

A slightly modified version of the indirect labeling protocol from The Institute of Genomic Research (TIGR, Rockville, Maryland, USA) was used for labeling with Cy5 and Cy3 dyes. The TIGR hybridization protocol was also slightly modified for the current study. Details have been described elsewhere [19].

### Microarray scanning and image quantification

The scanner calibration slide was scanned from 150 V to 1000 V PMT gains at an interval of 50 V under the same laser power setting and at a resolution of 10 μm on a GenePix 4000 A scanner (Axon Instruments, Inc., Union City, California, USA), resulting in 18 scans of the same slide for each channel. A 16-bit TIFF image was acquired for each scan and quantified by using GenePix 4.0 software (Axon Instruments, Inc.) under the same procedures and parameter settings [7]. Data from repeated scans under the same PMT gain before and after the whole scanning process showed minimal signal degradation.

### Microarray data analysis

Microarray data were stored in ArrayTrack, a database and software system developed by the FDA's National Center for Toxicological Research for the management, analysis, and interpretation of DNA microarray data [20,21]. Additional calculations were performed within S-Plus 6.1 (Insightful Corp., Seattle, Washington, USA), JMP 5.0.2 (The SAS Institute, Carry, North Carolina, USA), and DMVS 2.0 (Chipscreen Biosciences Ltd., Shenzhen, China).

### Estimation of log ratios based on intensities

The fluorescence intensity data acquired from different PMT gains for the various concentration series allowed us to generate ratio data in a comprehensive way. First, Cy5 and Cy3 PMT gains are paired in 324 (18 times 18) ways. Second, each concentration series for one channel is paired with all concentration series of the other channel to generate many combinations of varying Cy5 and Cy3 concentrations, hence various ratios. To minimize the impact of saturated and undetectable spots on the accuracy of calculations, we have arbitrarily excluded the nine lowest concentration series for which the signal intensities were below the detection limit for most PMT gains. The five highest concentration series for which the signal intensities were saturated for most PMT gains were also excluded. Thus, 14 concentration series remained for each channel, resulting in 196 (14 times 14) possible combinations of Cy5 and Cy3 concentrations. Therefore, we obtained a log ratio matrix of 196 rows and 324 columns, corresponding to different combinations of concentrations and PMT gains, respectively.

### Calculation of standard (true) log ratios

The standard (true) log ratios, StlgR, for the 196 concentration combinations discussed above were directly calculated from the spotted dye concentrations, instead of from measured fluorescence intensities.

## Results

### Characteristics of the calibration curves of Cy5 and Cy3 channels

**Figure 2:**
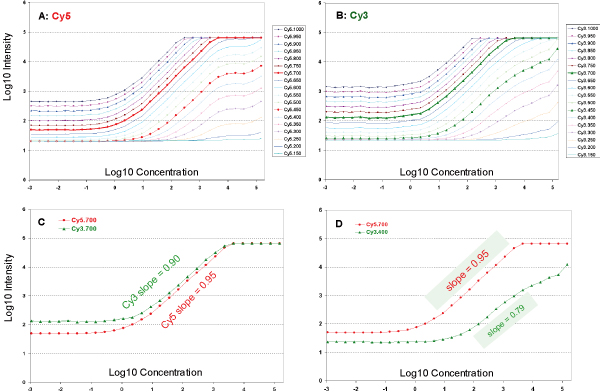
**Calibration curves under different PMT gains. **X-axis: log10 concentration, Y-axis: log10 fluorescence intensity. A: Cy5 dye; B: Cy3 dye. Representative calibration curves are presented in C (Cy5 and Cy3 channels are scanned under the same PMT gain of 700 V) and D (the Cy5 and Cy3 channels are scanned at 700 V and 400 V, respectively). The range of linear response between dye concentration and fluorescence intensity depends on the PMT gain, so does the slope of the linear response range. There is also a difference between Cy5 and Cy3 in terms of the spread and slope of the linear range. Each data point represents the averaged log intensity of the 12 repeats with the same dye concentration.

A scanner calibration slide with the layout, shown in Figure 1, was used to examine the characteristics of the calibration curves for Cy5 and Cy3 as described in the Methods section. Calibration curves for each dye under 18 different PMT gains (from 150 V to 1000 V at an interval of 50 V) are shown in Figures 2A and 2B. Two representative calibration curves are shown in Figure 2C, where the PMT gain for both channels is set to 700 V, which appears to be in the center of the optimal range of gain setting for the Axon GenePix scanner used in this study [5,6]. Figure 2D shows the calibration curves for Cy5 and Cy3 scanned at a gain of 700 V and 400 V, respectively. Several observations regarding the characteristics of the calibration curves are worth noting.

First, at any given PMT gain for the same dye, the fluorescence intensity increases as the dye concentration increases, and there is a range within which the signal increases linearly with dye concentration. This range is defined as the linear dynamic range, which can be expressed in terms of concentration range or intensity range. However, at higher concentrations, and well before the digital saturation at the intensity of 65535 (2^16^-1) for a 16-bit scanner, the intensity no longer increases linearly with concentration. In addition, nonlinearity also occurs at lower concentrations and intensities.

Second, the linear dynamic range in terms of fluorescence intensity varies with PMT gain. For example, at lower PMT gains (*e.g*., 150 V–550 V), the linear range is narrower than that for higher PMT gains (*e.g*., 600 V–800 V). The linear range also narrows at high PMT gains (*e.g*., 850 V–1000 V) due to the saturation of spots with higher dye concentrations and a significant increase in background fluorescence intensity. Nonlinearity occurs at both higher and lower concentrations in a manner that heavily depends on the PMT gain setting.

Third, the slope of the linear part of the calibration curves varies for the same dye with different PMT gains; *i.e*., the slope is significantly lower when PMT gain is too low. For example, the slope for Cy3 is 0.90 at 700 V (Figure 2C) compared with 0.79 at 400 V (Figure 2D). It is worth noting that at lower PMT gains (*e.g*., <550 V for Cy5 and <500 V for Cy3), fluorescence intensity can hardly reach the level of digital saturation (65535) at the highest dye concentration on the calibration slide.

Fourth, there are inherent differences in the calibration curves for Cy5 and Cy3 at the same PMT gain setting. Generally, the slope for Cy5 is somewhat higher than that for Cy3 at the same PMT gain setting. For example, the slopes for Cy5 and Cy3 are 0.95 and 0.90, respectively, for a PMT gain of 700 V (Figure 2C), under which the inherent differences between Cy5 and Cy3 appear to be the smallest.

Fifth, the background fluorescence intensity differs significantly for Cy5 and Cy3 under the same PMT gain, and for the same dye under different PMT gains. We consider background as the fluorescence intensity level that does not change with dye concentration. The differences in background levels appear to be due to the inherent differences of the two photomultiplier tubes for the Cy5 and Cy3 dyes.

The nonlinearity in calibration curves, the differences in the spread and slope of the linear range of the calibration curves, and the differences in background level of the two dyes have important implications as to the reproducibility and accuracy of fluorescence intensities and the calculated gene expression ratios.

### Reproducibility of log intensities

**Figure 3:**
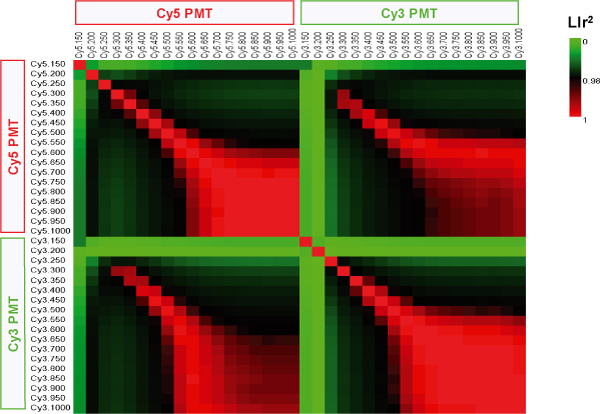
**Reproducibility of log intensities. **This is a color-image presentation of a 36 by 36 square matrix. Each square block represents the squared Pearson correlation coefficient of log intensities (LIr^2^) for two series of intensity data scanned at two PMT settings. The diagonal represents self-self correlation. Red color means higher correlation, whereas green indicates lower correlation. Only 14 concentration series are used in the calculation of LIr^2^.

The reproducibility of fluorescence data acquired under different PMT gains is measured by the squared Pearson correlation coefficient of log intensities (LIr^2^). To minimize the impact of spots that are saturated or below detection limits, five dilution series at the high concentration end and nine dilution series at the low concentration end were excluded for the following calculations. Therefore, only 14 concentration series in the middle, covering a dye concentration difference of 2^14-1 ^= 4096-fold, were used in the calculation of LIr^2^. The pair-wise correlation matrix (36 by 36, 18 PMT gains for Cy5 and 18 PMT gains for Cy3) is represented in Figure 3, as a color-coded image map [22]. Each block represents the LIr^2 ^value for the two series of log intensity acquired under two PMT gains. The diagonal represents self-self correlation. Red color means higher correlation, whereas green indicates lower correlation. It is clear that correlation is lower for intensities acquired under lower PMT gains (*e.g*., <600 V); a small change in PMT gain at the lower PMT range will result in a significant decrease in the correlation of the fluorescence intensity. On the other hand, correlation is much higher for intensities acquired under higher PMT settings (*e.g*., >600 V). This trend is observed both for intensity data acquired under different PMT gains within the Cy5 (upper-left quarter) or Cy3 (lower-right quarter) channel individually, and also for the correlation between the two channels (lower-left or upper-right quarters). Similar graphs were obtained by excluding different numbers of concentration series (data not shown). Figure 3 demonstrates the importance of scanning microarray slides within the optimal range of PMT gains (*e.g*., 600 V–800 V) and of keeping the scanning PMT gain as consistent as possible, in order to generate reproducible fluorescence intensity data during a microarray study. Inconsistent intensity measurement due to a PMT gain difference results in inconsistent ratios.

### Reproducibility of log ratios

**Figure 4:**
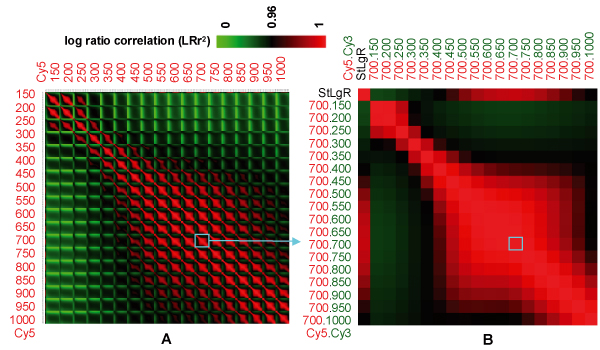
**Reproducibility of log ratios. **A: Correlation matrix for 324 pairs of Cy5/Cy3 gains; B: Correlation matrix for Cy5 gain at 700 V and 18 gains for Cy3. StLgR refers to the standard log ratios calculated from the spotted Cy5 and Cy3 concentrations on the scanner calibration slide.

The 196 by 324 log ratio matrix, as calculated by following the procedure described in the Methods section, represents estimated log ratios for 196 (14 times 14) Cy5/Cy3 concentration combinations under 324 (18 times 18) Cy5/Cy3 PMT gain combinations. It allows investigation of effects of PMT gain setting on the reproducibility and accuracy of log ratios calculated from fluorescence intensity. From the log ratio matrix, a 324 by 324 matrix of squared Pearson correlation coefficient of log ratios (LRr^2^) was calculated column-wise. The correlation between different pairs of Cy5/Cy3 gains varies dramatically as shown in Figure 4A. Numbers shown in Figure 4A represent Cy5 gains, each of which is paired with a series of 18 different PMT gains for Cy3 (from 150 V to 1000 V). Figure 4B shows a sub-matrix for Cy5 gain of 700 V and all 18 PMT gains for Cy3. The 700 V/700 V pair appears to be in the center of the PMT gains whereby some degree of gain adjustment may be tolerated without dramatic impact on LRr^2^.

### Accuracy and underestimation of log ratios

**Figure 5:**
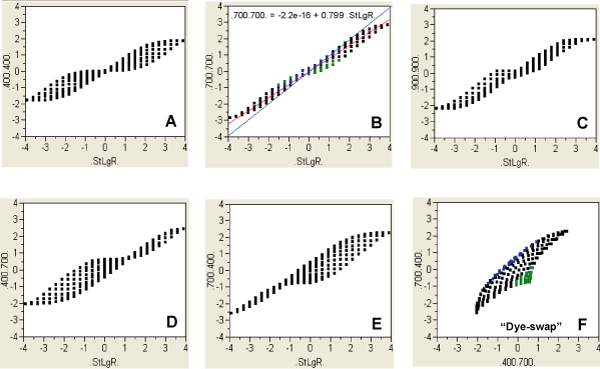
**Accuracy and underestimation of log ratios. **The correlation of true log ratios (StLgR) versus log ratios estimated by fluorescence intensities under different combinations of Cy5/Cy3 PMT gain settings reveals systematic ratio bias. Intensity data are mean-zero normalized for each individual channel at each PMT gain. The log ratios are estimated from different Cy5/Cy3 PMT gain combinations; A: 400 V/400 V; B: 700 V/700 V; C: 900 V/900 V; D: 400 V/700 V; and E: 700 V/400 V. Figure 5E corresponds to a dye-swap pair in which the Cy5/Cy3 PMT gains are set to 400 V/700 V and 700 V/400 V for the X- and Y-axes, respectively.

The log standard ratios (StLgR) were calculated directly from the corresponding concentrations from the spotted dilution series of Cy5 and Cy3 on the scanner calibration slide and thus report the "true" log ratio values. The correlation between StLgR and log ratios estimated from fluorescence intensities heavily depends on the Cy5/Cy3 PMT gain (top row of Figure 4B). Representative scatter plots showing the relationship between StLgR and estimated log ratios are given in Figures 5A–E. The log ratios calculated from intensities obtained at 700 V/700 V (Figure 5B) are closer to StLgR than those at other gains (*e.g*., 400 V/400 V, Figure 5A). Notably, there is a significant bias in log ratios calculated from intensities: absolute log ratios are dramatically underestimated compared to truth, in particular for large fold changes. The severity of ratio underestimation depends on the PMT gains, and the 700 V/700 V gain for Cy5/Cy3 appears to show the least degree of ratio underestimation. Ratio underestimation is a well-known phenomenon of microarray data [14,23]. Our results are consistent with such observations. Ratio underestimation can be partially attributed to the nonlinearity of the calibration curves.

### Intensity-dependence of anti-correlation

**Figure 6:**
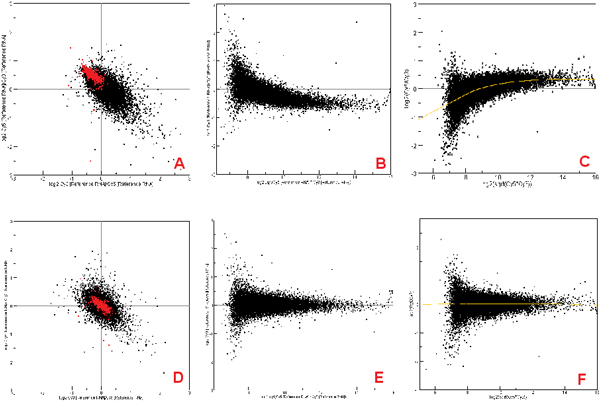
**Experimentally observed anti-correlation of log ratios for dye-swap replicates. **A: Anti-correlation of dye-swap replicates (self-self hybridizations) before Lowess normalization; B and C: MA plots for dye-swap pair before Lowess normalization; D: Correlation of dye-swap replicates after Lowess normalization; E and F: MA plots for dye-swap pair after Lowess normalization. Spots colored in red are of higher average intensity.

Dye-swap replicates are routinely performed in two-color platforms for correcting "dye-bias" [24-26]. In performing such experiments, we observed a characteristic, anti-correlation, which is strongly intensity-dependent (Figure 6A). Shown in MA (or RI) plots [11-13], this anti-correlation corresponds to an intensity-dependence of log ratio bias for each replicate of the dye-swap pair (Figures 6B and 6C). Shown in Figure 6D is the log ratio correlation for the dye-swap pair after Lowess normalization, and the corresponding MA plots are shown in Figures 6E and 6F. The intensity-dependence of the anti-correlation of log ratios is less profound after Lowess normalization (Figure 6D) compared to mean normalization (Figure 6A). Note that colored in red are genes with the highest intensity and their log ratios are significantly deviated from 0 in an anti-correlation (Figure 6A); whereas the log ratios for the same subset of genes clouded around 0 (Figure 6D). The examples shown in Figures 6A–F were from two self-self hybridizations with universal mouse reference RNA samples from Stratagene (La Jolla, California, USA). The slides were scanned under Cy5/Cy3 PMT gains of 700 V/600 V on an Axon GenePix 4000B scanner. When two samples with significant biological differences are compared, the corresponding dye-swap replicates show a characteristic, intensity-dependent X-shaped anti-correlation in which some genes show a positive correlation, whereas a significant number of genes show an anti-correlation (data not shown).

### Simulation of a dye-swap experiment

Like many dose-response curves observed in biological sciences and the calibration curves of many analytical instruments, for a microarray scanner the calibration curves (Figure 2) that show the relationship between log fluorescence intensity (*I*) and log dye concentration (*c*) may be reasonably fitted into a Sigmoid function:



where *θ *defines the spread and slope of the linear range of a Sigmoid curve and the "background" level; *D *is the upper limit of the dynamic range and is set to 3 in this study. The simulated data with different *θ *values can be reasonably seen as fluorescence intensities obtained from different PMT gains for the same dye or from the same PMT gain setting for two different dyes.

**Figure 7:**
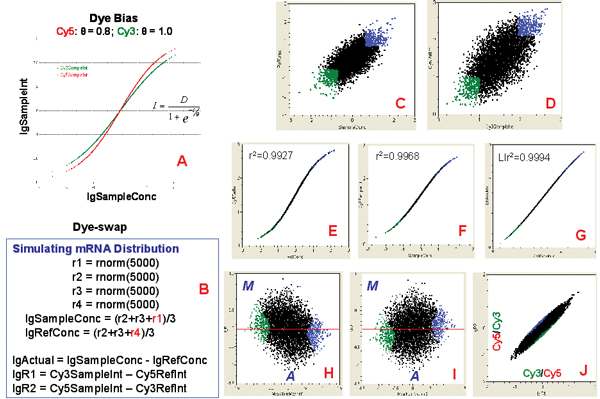
**Simulation of the characteristics of dye-swap replicates. **A: Dye bias is simulated by a difference in *θ *of the Sigmoid function; B: Dye-swap pairing; C: log mRNA concentrations for the two samples ("Sample" and "Ref"); D: Scatter plot of log intensities for one replicate of the dye-swap pair; E: Calibration curve for Cy5 ("labeled" with "Ref"); F: Calibration curve for Cy3 ("labeled" with "Sample"); G: log intensity correlation for the same sample ("Sample") labeled with two dyes in the dye-swap pair; H: MA plot for "Ref" labeled with Cy5 and "Sample" labeled with Cy3; I: MA plot for "Ref" labeled with Cy3 and "Sample" labeled with Cy5; J: log ratio correlation of the dye-swap pair. Spots colored in green are of lower average intensity, whereas spots colored in blue are of higher average intensity.

Figures 7A–J show the simulation results corresponding to *θ *values of 0.8 and 1.0 for Cy5 and Cy3, respectively. This difference in *θ *simulates the degree of dye bias (Figure 7A). The log mRNA concentrations for the two RNAs ("Sample" and "Ref") are assumed to follow a normal distribution (Figure 7B) and to have a Pearson correlation coefficient of ~0.67 with 5000 genes (Figure 7C). The intensity data calculated from this Sigmoid function can be regarded as log intensity data that have a range between 0 and 3 and a mean (and median) value of 1.5 (Figure 7D). Each RNA sample can be "labeled" with either Cy5 or Cy3 in a dye-swap pair. The calibration curves for Cy5 and Cy3 are shown in Figures 7E and 7F, corresponding to the labelling of "Ref" RNA with Cy5 and "Sample" with Cy3, respectively. Figure 7G illustrates the log fluorescence intensity correlation corresponding to the same RNA "Sample" labeled with different dyes. It is worth noting that although the nonlinearity of the calibration curves is severe (Figures 7E and 7F), the log intensity correlation for the same RNA sample in a dye-swap is much less profound (Figure 7G). The MA plots for the dye-swap pair show a mild intensity-dependence of log ratios (Figure 7H and 7I). The intensity-dependent anti-correlation of the dye-swap pair is also mild, but obvious (Figure 7J).

### Comparison of mean and Lowess normalization

**Figure 8:**
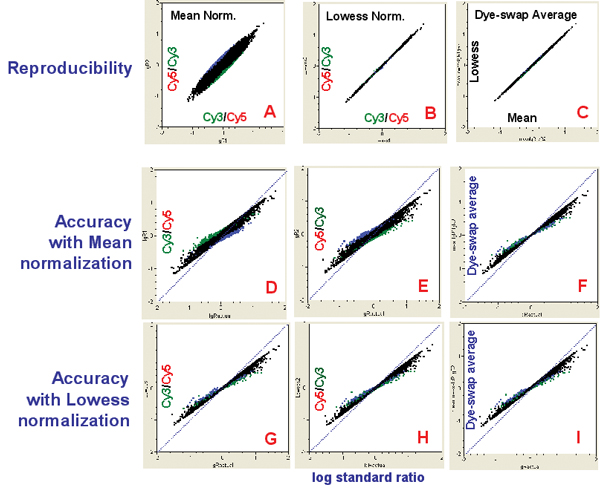
**Effect of normalization on the reproducibility and accuracy of log ratios (simulated data). **A: log ratio correlation of dye-swap pair after mean normalization; B: log ratio correlation of dye-swap pair after Lowess normalization; C: Correlation of averaged log ratios of dye-swap pairs after mean and Lowess normalization. For D-I, the X-axis represents the log standard ratios directly calculated from concentrations. The Y-axis is represented as follows: D and E: log ratios for dye-swap pair (after mean normalization); F: Averaged log ratios of dye-swap pair (after mean normalization); G and H: log ratios for dye-swap pair (after Lowess normalization); I: Averaged log ratios of dye-swap pair (after Lowess normalization). Spots colored in green are of lower average intensity, whereas spots colored in blue are of higher average intensity.

The effectiveness of two normalization methods (*i.e*., mean-intensity scaling and Lowess) on the reproducibility and accuracy of log ratios was assessed using the simulated dye-swap dataset discussed above. The results are shown in Figure 8 in terms of reproducibility and accuracy. The log ratio reproducibility for mean (Figure 8A) and Lowess (Figure 8B) normalization is illustrated in scatter plots. While mean normalization (Figure 8A) has no effect in correcting intensity-dependence of log ratio bias seen in Figures 7H–J, Lowess effectively removes the intensity-dependence of log ratio bias or anti-correlation and makes the dye-swap pair much more consistent to each other (Figure 8B) compared to Figure 7J. However, when the dye-swap pair is averaged after normalization, the difference between mean and Lowess normalization is minimal (Figure 8C).

When accuracy (*i.e*., the closeness between log estimated ratios and the log standard ratios) is considered (Figures 8D–I), the effectiveness of both mean and Lowess normalization appears questionable: ratio bias (underestimation) remains. Simulation results were also obtained by modeling more severe dye-bias with a larger difference in the *θ *parameter for the two dyes. The intensity-dependence of ratio bias and anti-correlation of the dye-swap pair became more dramatic (data not shown), and the effectiveness of the mean and Lowess normalization methods in correcting ratio bias remains minimal.

### Concentration-based ratio calculation for correcting ratio bias

The intensity-dependent ratio bias and the anti-correlation appear to be a result of the nonlinearity of the calibration curves and the calculation of ratios from dividing the measured fluorescence intensities from the two channels. That is, the calculated fold changes obtained by directly dividing measured intensities do not accurately reflect the true fold difference in concentration.

**Figure 9:**
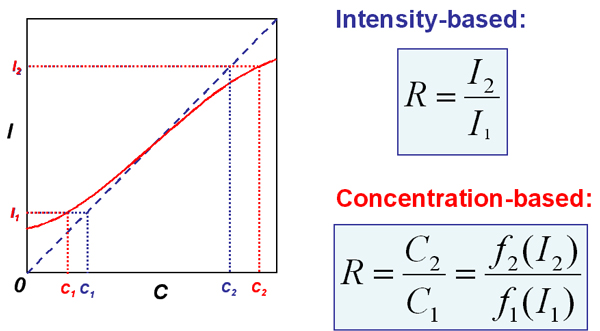
**Intensity-and concentration-based ratio calculation. **Intensity-based ratio calculation is accurate only when the calibration curve (I~C) is a linear and pass the origin (the dashed blue line). When the calibration curve is nonlinear (red curve), intensity-based ratio calculation underestimate the true concentration differences. Such an underestimation of concentration difference is universal for both two-color and one-color platforms. For one-color platform, the calibration functions *f*_*1 *_and *f*_*2 *_are the same, whereas for two-color platform, *f*_*1 *_and *f*_*2 *_reflect the differences of the two dyes as discussed in this study.

Instead of calculating the ratio by directly dividing the two fluorescence intensities from the two channels, we propose a method for calculating the ratio in the hope of circumventing ratio bias (Figure 9). The essence is to divide the concentrations (instead of intensities) estimated from the calibration curves for both channels. For each channel under a given PMT gain, a calibration function *c *= *f*(*I*) can be derived by using the calibration data. For a real experiment, each intensity value can be transformed by the calibration function *f *into an estimated dye concentration. Then, the ratio is obtained by dividing the two concentration values estimated from the two intensities for the same spot.

**Figure 10:**
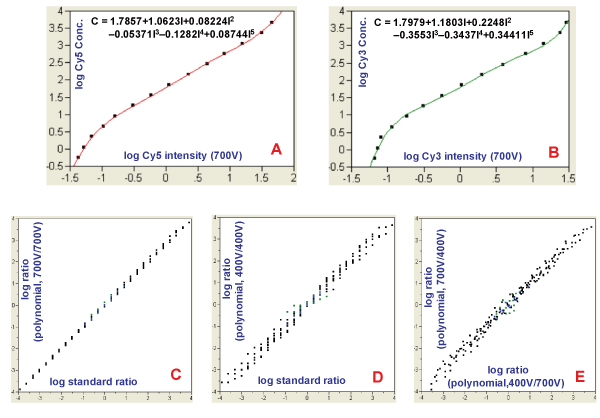
**Correction of ratio bias by calculating ratios from the estimated concentrations from the calibration curves. **A: Polynomial fitting of *c *= *f*(*I*) for Cy5 at 700 V gain; B: Polynomial fitting of *c *= *f*(*I*) for Cy3 at 700 V gain; C: log standard ratios versus log ratios calculated from concentrations estimated from the calibration curves (Cy5/Cy3 PMT gains at 700 V/700 V); D: log standard ratios versus log ratios calculated from concentrations estimated from the calibration curves (Cy5/Cy3 PMT gains at 400 V/400 V); E: log ratios calculated from concentrations estimated from the calibration curves under Cy5/Cy3 PMT gains at 400 V/700 V and 700 V/400 V, respectively. Data were from the scanner calibration slide. The effectiveness of concentration-based ratio calculation is more obvious by comparing Figures 10C, 10D and 10E to Figures 5B, 5A and 5F, respectively.

In this study, for each dye under a given PMT gain, a 5-term polynomial fitting equation was derived (Figures 10A and 10B) to represent the calibration function. The concentrations were estimated from the calibration functions and used for calculating ratios. The concentration-based ratios from the dataset obtained on the calibration slide are much closer to standard ratios (Figure 10C versus Figure 5A, Figure 10D versus Figure 5B), and ratios for the 700 V/700 V gains (Figure 10C) are more accurate than those for 400 V/400 V gains (Figure 10D). The "dye-swap" replicates are also much closer to each other (Figure 10E versus Figure 5F). Concentration-based ratio calculation effectively corrected ratio bias, ratio underestimation, and anti-correlation. Other functions (including a Sigmoid function) were also used to fit the calibration curves with similar effectiveness (data not shown).

## Discussion

We systematically assessed the characteristics of the calibration curves for Cy5 and Cy3 under 18 different PMT gain settings (Figures 2A–D) by using a scanner calibration slide spotted with pure fluorescent dyes. This approach enabled the separation of the effects of intrinsic characteristics of the two dyes (and the corresponding photomultiplier tubes for signal detection) from other experimental factors such as labeling and hybridization. The implications of the characteristics of the calibration curves have been demonstrated in terms of reproducibility and accuracy of log intensities and log ratios.

### PMT gain setting

Our analysis of data from the scanner calibration slide and simulation revealed marked sensitivity of PMT gain setting on DNA microarray reproducibility and accuracy. The sensitivity strongly suggests an essential need to minimize the impact of nonlinearities for accurate measurement of differential gene expression. For example, the optimal PMT range and calibration behavior of the scanner should be well determined. Furthermore, all slides within the same study should always be scanned within the optimal PMT gain range (*e.g*., 600 V–800 V) where linearity is maximized. Preferably, slides in a study should be scanned at consistent PMT gain. For the scanner used in this study, a PMT gain at 700 V appears to be in the center of the optimal range, and small adjustment within a certain range (*e.g*., +/- 50 V) appeared to be acceptable. To minimize the difference between Cy5 and Cy3, PMT gains for the two channels should be set in a way so that the calibration curves for the two channels are as close as possible. A microarray experiment well performed in all early steps such as sample preparation, cDNA or cRNA synthesis, dye labeling, and hybridization could be compromised if the slides are scanned at non-optimal and different PMT gains. A practice of fixing PMT gain in the optimal range has not always been followed because the adjustment of PMT gains has been made very easy for the user [7] and sometimes encouraged by the vendor. Consequently, we reason that the scanners and theirs parameter settings might have significantly contributed to the lack of reliability of microarray data. The optimal range of the PMT gains for each channel of a scanner should be well-defined.

### Possible causes of ratio underestimation

The accuracy of Affymetrix chips and customized cDNA microarrays have been assessed by comparing detected ratios to those from qRT-PCR [23]; both platforms consistently underestimate ratios. Hekstra *et al*. [27,28] and Held *et al*. [29] addressed the problem of sequence-specific response of fluorescent signal as a function of concentration, and proposed ways to correct ratio underestimation for genes with high-fold changes observed in Affymetrix chips based on Langmuir adsorption and free-energy calculations, respectively.

Although ratio underestimation has become a commonly recognized feature of microarray technology, the exact causes have not been fully understood. Our results demonstrate that nonlinearity of the calibration curve is one of the causes of ratio underestimation, and the severity of ratio underestimation is closely related to the severity of the nonlinearity of the calibration curves under different PMT gains (Figures 5A–E). To minimize ratio underestimation, the linear dynamic range of the calibration curves of the scanner should be maximized and background intensity should be minimized. We noted that background-subtracted intensity improves the linear dynamic range of the calibration curve (data not shown).

Another important cause of ratio underestimation is non-specific binding, as explained by the following equation:



where *R *is ratio; *I *is the fluorescence intensity; and *s *and *ns *stand for specific and non-specific binding, respectively. When the intensity from both channels (1 and 2) are significantly contributed by non-specific binding, the calculated ratio will significantly deviate from the true ratio of *I*^*s*^_*1*_*/I*^*s*^_*2*_, based on a reasonable assumption that the contribution of the non-specific binding for the two channels (I^*ns*^_1 _and I^*ns*^_2_) are similar. For up-regulated genes (*i.e*., *I*^*s*^_*1 *_> *I*^*s*^_*2*_), the calculated ratio (a number > 1) will be smaller than *I*^*s*^_*1*_*/I*^*s*^_*2*_. For down-regulated genes (*i.e*., *I*^*s*^_*1 *_<*I*^*s*^_*2*_), the calculated ratio (a number < 1) will be greater than *I*^*s*^_*1*_*/I*^*s*^_*2*_. In both cases, there is an underestimation of the absolute log ratio. One extreme situation is that the contribution of non-specific binding is overwhelming compared to that of specific binding, thus leading to a ratio close to 1. This phenomenon has been experimentally observed in our laboratories when a non-optimized hybridization buffer from a commerical source was used (Han T *et al*., data not shown).

### Intensity-dependence of anti-correlation and ratio bias

The main reason for anti-correlation between dye-swap replicates is the inherent differences in the calibration curves for the two dyes (Cy5 and Cy3). The nonlinearity of the calibration curves, in particular under a PMT gain setting at non-optimal range, is one cause for anti-correlation. A pronounced difference in Cy5 and Cy3 background has been observed [8-10] and can be another cause for ratio bias. What has been shown in our results is likely a combination of nonlinearity and background difference.

Gene-specific bias in binding affinity to the two dyes has been suggested to be a cause of ratio bias for some genes [24,25]. Our data provide an alternative explanation to the phenomenon of intensity-dependence of ratio bias [11-13] as a result of the inherent differences in calibration curves of the two dyes where no labeling or hybridization steps are involved.

### Correction of ratio bias

We demonstrate that normalization methods (including Lowess), while improving reproducibility, are not effective in reducing ratio bias from the truth. It appears that normalization methods and the averaging of dye-swap replicates effectively "hide" rather than reduce the problems related to ratio bias.

Strategies for correcting ratio bias by extending the dynamic range have been proposed [14-18], but such a procedure has not yet been adopted for routine use in microarray practice. Furthermore, ratio underestimation is still recognizable, *e.g*., after the Masliner correction (see Figure 2 of reference [14]). A perfect correlation in intensity (*e.g*., under the same PMT gain for the same dye) does not correct the intrinsic nonlinearity of the calibration curves (intensity versus concentration correlation). Rather, intensity correlation "hides" the nonlinearity of the calibration curves (Figure 7G versus Figures 7E and 7F). Therefore, the problems of nonlinearity observed in this study on the two-color platform largely apply to one-color platforms.

To effectively solve the problem of ratio bias due to nonlinearity in the calibration curves, we propose using concentration instead of intensity for ratio calculation. While this approach appears promising, a fundamental question is whether the calibration curves for different genes are similar enough for establishing a gene-independent calibration function, *c *= *f*(*I*); or whether it is feasible to obtain individual calibration curves for all the genes on a microarray. We are actively investigating this issue.

### Standards for the calibration and validation of microarray scanners

The reliability of microarray data cannot be better than that of the microarray scanner. Universal standard (or reference) materials need to be established for calibrating and validating microarray scanners. The performance of a microarray scanner should be routinely checked by standard materials like the calibration slide used in the study. The user should be made aware of the implications of the changes of scanner settings (*e.g*., PMT gain and laser power) so that variability due to the scanner can be minimized and the true biological information can be reliably obtained by microarray technology. More studies on the calibration and validation of microarray scanners and the correction of the resulting data are warranted, as are guidelines on the proper use of microarray scanners.

## Conclusion

Our results demonstrate the substantial impact of the PMT gain setting of a scanner on the reproducibility and accuracy of log ratios estimated by microarray technology resulting from the inherent characteristics of the two dyes under different PMT gains. Our data provide rational explanations to several experimental observations such as intensity-dependence of ratio bias, underestimation of ratio, and anti-correlation of dye-swap replicates. A concentration-based ratio calculation method is proposed for correcting ratio bias and underestimation. More studies on the effect of scanner settings on microarray data quality are warranted, and reference materials should be established for the calibration and validation of microarray scanners. Our results show that the effectiveness of normalization methods (including Lowess) in correcting ratio bias from the truth is very limited. The merits of various methods for the normalization, correction, and analysis of microarray data must be objectively assessed by using calibrated reference datasets so that not only reproducibility, but also accuracy, can be evaluated [3,30].

## Authors' contributions

LS had the original idea on the method and performed all experimental design, data analysis and simulations, and wrote the manuscript. WT, ZS, HF, SCH, HH and QX were involved in discussions on the data analysis and verified some of the calculations. JH, RKP, FWF, FMG and LG provided additional insights regarding issues on scanner calibration and validation. TH, WSB and JCF conducted hybridizations using in-house spotted microarrays and acquired the data presented in Figure 6. WSB also scanned another calibration slide on an Axon GenePix 4000B scanner under various PMT gains and three laser power settings (data not shown). ZAX conducted experiments with Agilent oligo microarrays and provided additional information on the characteristics of the Cy5 and Cy3 dyes. WT, RKP, JH, LG, JJC, RGP and JCF assisted with writing the manuscript. All authors participated in the design of the study and approved the final manuscript.
